# Eat to reproduce: a key role for the insulin signaling pathway in adult insects

**DOI:** 10.3389/fphys.2013.00202

**Published:** 2013-08-07

**Authors:** Liesbeth Badisco, Pieter Van Wielendaele, Jozef Vanden Broeck

**Affiliations:** Department of Animal Physiology and Neurobiology, Research Group of Molecular Developmental Physiology and Signal TransductionKU Leuven, Leuven, Belgium

**Keywords:** insulin signaling pathway, neuropeptides, lipophilic hormones, nutritional status, female insect reproduction

## Abstract

Insects, like all heterotrophic organisms, acquire from their food the nutrients that are essential for anabolic processes that lead to growth (larval stages) or reproduction (adult stage). In adult females, this nutritional input is processed and results in a very specific output, i.e., the production of fully developed eggs ready for fertilization and deposition. An important role in this input-output transition is attributed to the insulin signaling pathway (ISP). The ISP is considered to act as a sensor of the organism's nutritional status and to stimulate the progression of anabolic events when the status is positive. In several insect species belonging to different orders, the ISP has been demonstrated to positively control vitellogenesis and oocyte growth. Whether or not ISP acts herein via a mediator action of lipophilic insect hormones (ecdysteroids and juvenile hormone) remains debatable and might be differently controlled in different insect orders. Most likely, insulin-related peptides, ecdysteroids and juvenile hormone are involved in a complex regulatory network, in which they mutually influence each other and in which the insect's nutritional status is a crucial determinant of the network's output. The current review will present an overview of the regulatory role of the ISP in female insect reproduction and its interaction with other pathways involving nutrients, lipophilic hormones and neuropeptides.

## Introduction

In order to maintain the existence of a given species and to pass on the genetic material that defines the species, all living organisms must be capable of producing viable offspring, in a process called “reproduction.” As in many other animals, the embryos of most insect species develop within an egg, externally from the mother. It is therefore of crucial importance that the egg contains the necessary energy (nutrients), hormones and other components that are indispensable for embryonic development. These essential components are incorporated during oocyte development in the female insect's ovary. Synthesis and incorporation of these components require from the female a lot of energy, which she can only acquire by means of her nutritional input or by reallocation of previously stored energy-rich compounds. Although studied to a lesser extent, it is obvious that also in males the nutritional status is determinative for the development of viable sperm cells.

In an evolutionary conserved mechanism, increased insulin production and signaling—as a response to a positive nutritional status—tends to stimulate the start and progress of several anabolic processes, supporting growth (juveniles) and reproduction (adults). Multiple studies in different metazoan species have indeed demonstrated that not only the insulin-related peptides are evolutionary conserved, but also the components of their signaling pathway. As in other Metazoa, the insulin signaling pathway (ISP) is believed to exert a crucial role in a number of fundamental and interrelated physiological processes in insects (Claeys et al., 2002; Wu and Brown, 2006). In adult insects, the acquired nutritional input is processed and results in specific outputs, such as the production of mature gametes. With a specific focus on female reproductive physiology, the current review aims to illustrate the indispensable role of the ISP in this input-output transition.

## The insect insulin signaling pathway (ISP)

**Figure 1:**
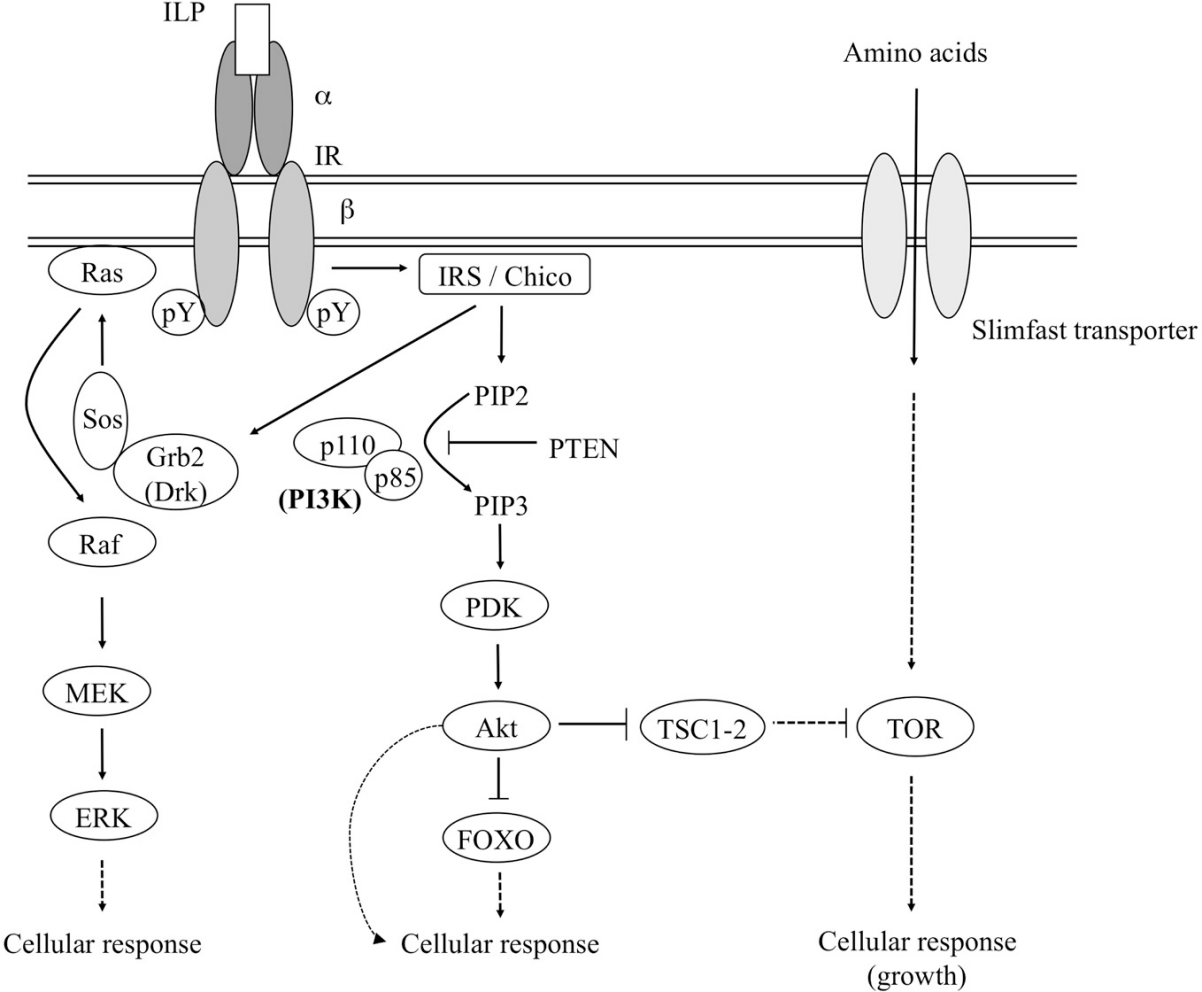
**Simplified schematic representation of the insulin and TOR signaling pathways as they have been described in mammals and for which orthologous components have been described in *D. melanogaster* and some other insects**. For a detailed overview of the pathway components the reader is referred to the text. Dashed lines represent indirect interactions. Abbreviations: ILP, insulin-like peptide; IR, insulin receptor; IRS, insulin receptor substrate; Grb2, growth factor receptor bound protein-2 [the *Drosophila* ortholog of which is termed the “downstream of receptor kinase” (Drk)]; Sos, son of sevenless; MEK, mitogen-activated ERK-activating kinase; ERK, extracellular signal regulated kinase; PI3K, phosphatidylinositol-3-kinase; PIP2, phosphatidylinositol-4,5-bisphosphate; PIP3, phosphatidylinositol-3,4,5-trisphosphate; PTEN, phosphatase and tensin homologue; PDK, phosphoinositide-dependent protein kinase; FOXO, forkhead box-related transcription factors, class O; TSC1-2, tuberous sclerosis 1-2 complex; TOR, target of rapamycin. [Figure adapted from Claeys et al. (2002).]

The ISP is evolutionary conserved and has been functionally demonstrated in diverse protostomian and deuterostomian lineages (Tatar and Yin, 2001; Claeys et al., 2002; Burnell et al., 2005; Sherwood et al., 2006; Wu and Brown, 2006; Blumenthal, 2010; Fontana et al., 2010; Kawada et al., 2010; Fujisawa and Hayakawa, 2012). In Figure 1 a schematic representation is given of the insulin signaling pathway as it has been described in mammals and for which orthologous components were identified from *Drosophila melanogaster* and other insects. The ISP agonists in insects are generally termed “insulin-like peptides” (ILPs) or “insulin-related peptides” (IRPs). The insulin receptor (IR) is a transmembrane receptor tyrosine kinase (RTK) and consists of a dimer of two αβ-monomers. The α-subunits define the ligand binding specificity, whereas the β-subunits mediate the insulin(-like) signal to downstream cellular components. The IR makes use of the insulin receptor substrate (IRS) as an adaptor molecule to initiate the ISP (White, 1998). Upon binding of the hormone (insulin or a related peptide) to its receptor, the β-subunits undergo autophosphorylation at specific tyrosine residues. The activated RTK subsequently phosphorylates specific tyrosine residues of the IRS (Yenush et al., 1996). The *D. melanogaster* IR (DIR) gene encodes two DIR isoforms, one of which highly resembles the mammalian IR. The other isoform displays a C-terminal extension of about 300 amino acids that shows similarity to certain domains of the *D. melanogaster* IRS (which is termed Chico), and is also capable of activating down-stream proteins in an IRS-independent manner (Fernandez et al., 1995). Whether the extended IR isoform also occurs in non-drosophilid insect species remains to be investigated.

The activated IRS recruits downstream factors toward the receptor-IRS complex. The phosphorylated tyrosine residues interact with specific “Src-homology 2” (SH2) domains in the Grb2 (“growth factor receptor bound protein-2”) or phosphatidylinositol-3-kinase (PI3K) proteins (Blenis, 1993; Shepherd et al., 1998). [Src-homology domains are highly conserved non-catalytic structural domains that were initially described in the protein tyrosine kinase-encoding *src* oncogene. SH2 structures mediate high-affinity phosphotyrosine-dependent binding between proteins and are mostly involved in formation of signaling protein complexes at or near the plasma membrane (Shpakov and Pertseva, 2000).] Grb2 [the *Drosophila* ortholog of which is termed the “downstream of receptor kinase” (Drk) (Olivier et al., 1993)] and PI3K each initiate a separate signaling pathway, namely the Ras-MAPK (“mitogen activated protein kinase”) and PI3K/PKB (PI3K/protein kinase B) pathway, respectively.

Following the activation of Grb2/Drk, an IRS-Grb2/Drk-Sos (“Son of Sevenless”) complex is formed. Via Ras/Raf proteins, this complex activates the MEK/ERK (“mitogen-activated ERK-activating kinase/extracellular signal regulated kinase”) signaling pathway, which controls many diverse cellular processes, such as proliferation, differentiation and development. Since the MEK/ERK signaling pathway is involved in many cellular processes, several different, cooperating mechanisms are necessary to determine the final outcome (Shaul and Seger, 2007).

Recruitment of PI3K (which is a dimer of a catalytic (p110) and a regulatory (p85) subunit) results in formation of the IRS-PI3K complex. Subsequently, PI3K catalyzes synthesis of PIP3 (phosphatidylinositol-3,4,5-trisphosphate) from PIP2 (phosphatidylinositol-4,5-bisphosphate). However, PTEN (“phosphatase and tensin homologue”) can reverse this conversion and can again decrease the level of PIP3 in the cell. The “phosphoinositide-dependent protein kinase” (PDK) responds to the high PIP3 levels by recruiting the protein kinase B (PKB), which is also termed “Akt” (Alessi and Cohen, 1998; Shepherd et al., 1998). Akt (indirectly) affects—by phosphorylation—a number of downstream protein substrates, amongst which the TSC1-TSC2 (“tuberous sclerosis 1-2”) complex (Avruch et al., 2005). Phosphorylation of the TSC1-TSC2 complex abolishes its (indirect) inhibitory action on the “target of rapamycin” (TOR) (Oldham et al., 2000; Huang and Manning, 2009). Akt and TOR are considered the “master regulator kinases” of the PI3K/PKB pathway.

By phosphorylation of downstream proteins, Akt indirectly stimulates and prevents anabolic and catabolic processes, respectively. Amongst the Akt targets are the “forkhead-related” FOXO family of transcription factors (Kramer et al., 2002). FOXO proteins are negatively regulated by the insulin signaling pathway, because Akt-mediated phosphorylation of FOXO molecules prevents them from being translocated to the nucleus (Lin et al., 1997; Ogg et al., 1997; Brunet et al., 1999; Kops et al., 1999). FOXO proteins regulate transcription of genes involved in stress resistance, DNA and protein repair and control of cell cycle (Daitoku and Fukamizu, 2007; Hedrick, 2009). In addition, they are indispensable in an organism's response to starvation, since they promote conservation of energy or even catabolism (Kramer et al., 2008). The effective activity of these factors is an important determinant of an organism's lifespan.

TOR activation occurs either as a downstream event in the ISP or, ISP-independently, by the availability of amino acids. In the latter case, the cellular uptake of amino acids by the Slimfast transporter indirectly results in TOR activation. Therefore, the TOR and ILP signaling pathways are considered as nutritional sensors at the cellular and systemic level, respectively. Depending on the available nutritional energy, the TOR signaling pathway drives the cellular decision whether to use energy and nutrients or whether to conserve them. TOR in its activated form stimulates protein synthesis, lipid synthesis and further uptake of nutrients, whereas it inhibits autophagy. Cell growth is thus the main output of active TOR signaling (Hietakangas and Cohen, 2009). It is in this context worth mentioning another evolutionary conserved energy sensing factor, namely the AMP-activated protein kinase (AMPK). Although mainly studied in vertebrate species, AMPK has in *Drosophila* accordingly been demonstrated to be activated upon low nutrient levels, which go accompanied by an increased AMP:ATP ratio (Pan and Hardie, 2002). Mammalian AMPK was shown to inhibit TOR signaling, by phosphorylation of both TSC2 (Inoki et al., 2003) and the TOR scaffold protein, Raptor (Gwinn et al., 2008). Studies in the nematode *Caenorhabditis elegans* indicate that AMPK-mediated inhibition of TOR also takes place in invertebrate species (Fukuyama et al., 2012).

## The insect's nutritional status

### The fat body

The fat body is an organ unique to insects that is indispensable for storage and release of energy reserves. It is distributed throughout the animal's body, mainly around the gut and the reproductive organs. In addition to being a storage organ, the fat body also plays a crucial role in the release of nutrients, in the synthesis of hemolymph proteins, in the endocrine system [for instance, expression of insulin-like peptides has been demonstrated in the fat body of some insect species (Badisco et al., 2008; Okamoto et al., 2009)], in the immune system (Ferrandon et al., 2007) and in the detoxification of nitrogen metabolism (Scaraffia et al., 2005). Because of its loose nature, the fat body is maximally exposed to the hemolymph, which is crucial for the functions it exerts. In addition, it is a heterogeneous organ being regionally differentiated into different types of cells that each exert specific tasks (Roma et al., 2010). The adipocytes (also termed “trophocytes”) are the storage sites for carbohydrates, lipids and proteins and make out an important part of the fat body tissue. The size of the fat body is determined by the insect's life stage and is largely dependent on the amount of stored material. Information about the insect's nutritional status is thus not only available from the hemolymph, but also directly from the fat body.

**Figure 2:**
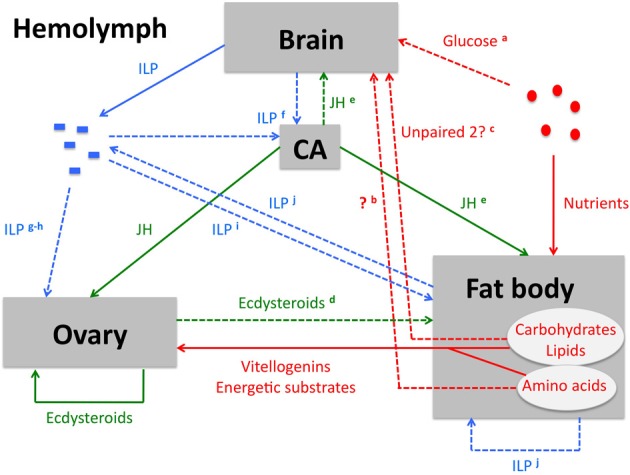
**Schematic overview of the key players in ISP-mediated control of female insect reproductive physiology**. Solid lines represent a consensus, a process that has been demonstrated in several insect orders. [It needs to be emphasized that the role of ISP in honeybee reproduction strongly deviates from the consensus. Therefore, information from the honeybee is not taken into account in this overview.] Dashed lines represent processes that have only been demonstrated in a limited number of insect species or orders, or that may possibly act more indirectly than suggested on the figure. These lines go accompanied by a remark, indicated by a superscript character. **Nutrient-related (signaling) pathways (red arrows):** Upon digestion of food in the midgut, nutrients are absorbed by the midgut cells and subsequently released into the hemolymph. They are either directly used by the tissues as a source of metabolic energy or as a substrate in anabolic reactions, or they are stored in fat body cells. The fat body's nutritional stores may then be mobilized for the production of vitellogenins, energetic substrates and other metabolic products that serve the process of oogenesis within the ovary. The TOR signaling pathway constitutes a conserved cellular nutrient (amino acid)-sensing system (Hietakangas and Cohen, 2009) and is therefore indispensable in the control of vitellogenin synthesis in the fat body. **^(a)^** Glucose availability stimulates the *in vivo* release of bombyxin from the silkworm brain, although a direct effect on the brain has so far not been proven (Masumura et al., 2000). Although the isolated fruit fly brain does not seem to release ILPs in response to glucose *in vitro* (Geminard et al., 2009), it is worth noting that some electrical properties of ILP producing brain cells appear to be affected by the glucose (Fridell et al., 2009; Kreneisz et al., 2010). **^(b)^** A hitherto unknown humoral factor that stimulates ILP release from the fruit fly brain is released from the fat body via a TOR-mediated response to the presence of amino acids (Colombani et al., 2003; Geminard et al., 2009). **^(c)^** Similarly, the cytokine-like factor Unpaired 2 is produced in the fat body in response to dietary fats and sugar. Remarkably, Unpaired 2 also appears to induce the release of ILP from the fruit fly brain. Although Unpaired 2 has so far not been demonstrated in the fruit fly hemolymph circulation *in vivo*, the authors of the corresponding report suggested that this protein may act as a humoral factor (Rajan and Perrimon, 2012). **Lipophilic hormone signaling (green arrows):** Ecdysteroids are in adult insects mainly synthesized by the gonads. In females they fulfill auto- and paracrine roles in ovary and oocyte development. In addition, ecdysteroid conjugates are stored in the eggs as an embryonic source of these lipophilic hormones. Juvenile hormone is produced by the CA. It stimulates vitellogenin production by the fat body, as well as vitellogenin sequestration by the developing oocytes. **^(d)^** In dipteran species, an endocrine function for the ecdysteroids in the regulation of vitellogenin synthesis has been demonstrated (Huybrechts and De Loof, 1977). An endocrine role seems to be attributed to ecdysteroids in *B. mori* too, since in this insect they are capable of stimulating ILP synthesis in the fat body (Okamoto et al., 2009, 2011). Remarkably, the decline of ecdysteroids appears to be crucial for termination of vitellogenesis in both *A. aegypti* and *B. mori*, indicating that the outcome of ecdysteroid action in reproductive physiology is stage-dependent and species-specific (Dhadialla et al., 1998; Swevers and Iatrou, 2009; Bryant and Raikhel, 2011). **^(e)^** In the beetle *T. castaneum* a stimulatory effect of JH on expression of some ILP genes in brain and fat body was shown (Sheng et al., 2011). **ILP signaling (blue arrows):** A stimulatory role of insulin signaling on JH biosynthesis has been shown in several insect orders, **^(f)^** although it is not clear whether ILPs are directly delivered to the CA by projections of the CC or whether they are received from the circulating hemolymph (Tu et al., 2005; Belgacem and Martin, 2006). **^(g)^** A stimulatory effect of ILPs on ovarian ecdysteroidogenesis has so far only been demonstrated in dipteran species (Tu et al., 2002; Brown et al., 2008; Wen et al., 2010). **^(h)^** Direct ILP-mediated positive control of oogenesis has hitherto only been shown in *D. melanogaster* (Drummond-Barbosa and Spradling, 2001; LaFever and Drummond-Barbosa, 2005; Richard et al., 2005) and *T. castaneum* (Parthasarathy and Palli, 2011). **^(i)^** Similarly, direct ILP-mediated stimulation of vitellogenesis has so far only been observed in Diptera (Roy et al., 2007; Gulia-Nuss et al., 2011) and in *T. castaneum* (Parthasarathy and Palli, 2011; Sheng et al., 2011). **^(j)^** In some insect species, ILP synthesis also occurs in the fat body (Badisco et al., 2008; Okamoto et al., 2009). In the desert locust, *S. gregaria*, the expression levels in this tissue are temporally regulated during the reproductive cycle (Badisco et al., 2008). It is therefore possible that ILP produced by the fat body also acts, at least in this insect species, as a paracrine messenger that signals information about the nutritional status within this tissue and stimulates vitellogenin production (Badisco et al., 2011). Abbreviations: CA, *corpora allata*; CC, *corpora cardiaca*; ILP, insulin-like peptide; JH, juvenile hormone; TOR, target of rapamycin.

Triglycerides have a higher caloric content per unit of weight than glycogen. Furthermore, a considerable source of water is released upon their oxidation. Therefore, the fat body has a higher capacity for lipogenesis from glucose than for glycogenesis, and accordingly lipids are the major components of the fat body (Inagaki and Yamashita, 1986; Zhou et al., 2004). A considerable part of an organism's amino acid reserves are stored as circulating storage proteins, such as hexamerins. These proteins are synthesized by the fat body and released into the hemolymph. When the amino acid reserves are needed, the storage proteins are taken up again by the fat body, in a receptor-mediated endocytotic process. Within the fat body cells, they are temporarily stored in protein granules. Subsequently, they are proteolytically degraded and serve as a source of amino acids (Haunerland, 1996). In all insect species, the period preceding vitellogenesis (i.e., yolk protein precursor production) is characterized by increased uptake of the storage proteins by the fat body, where this source of amino acids serves the synthesis of yolk proteins. Like growth, reproduction-related processes go accompanied with extensive protein synthesis, which is an energetically expensive process that depends upon the oxidation of specific compounds, such as carbohydrates and lipids. Therefore, strict (hormonal) control of storage and mobilization of energy reserves from the fat body is crucial for the correct progression of these reproductive anabolic processes. In addition, the fat body must be capable of integrating signals from other organs and those concerning the insect's nutritional status (Figure 2). [For a comprehensive review on the fat body, its biological functions and its regulation, we refer to Arrese and Soulages (2010).]

### The ISP acts as a sensor of nutritional status

Like all other animals, insects need to ingest food for the acquisition of energy and nutrients, in support of their metabolism. According to the physiological and developmental needs of the insect, the acquired nutrients can be differentially allocated to different organs, processes and/or metabolic pathways, enabling the insect to adjust its physiology according to its internal nutritional state. Nutrient sensing systems play an important role in this physiological adjustment process. An evolutionary conserved systemic nutrient sensor is the ISP. In general, this pathway is linked with the internal metabolic and nutritional state of metazoans and plays an important role in the induction of anabolic processes (Tatar and Yin, 2001; Burnell et al., 2005; Mukhopadhyay et al., 2006; Wu and Brown, 2006; Sim and Denlinger, 2008; Toivonen and Partridge, 2009; Fontana et al., 2010; Luedtke et al., 2010; Teleman, 2010).

Many reports confirm that also in insects, this general concept of ISP's role in controlling (certain aspects of) anabolism holds true. Nevertheless, clear functional differences have arisen between some of the ILPs of the different insect taxa, probably due to the long evolutionary history of the distinct insect orders, which has resulted in a variety of different life cycles, life history traits, feeding habits and feeding patterns. Moreover, gene duplication events that resulted in different numbers of paralogs in different taxa, might also have contributed to this [e.g., only 1 ILP (IRP) currently identified in locusts (Hetru et al., 1991; Badisco et al., 2008), while 8 ILPs have been demonstrated in *Drosophila* (Vanden Broeck, 2001; Colombani et al., 2012; Garelli et al., 2012; Nassel, 2012) and more than 30 seem to occur in *Bombyx mori* (Iwami, 2000; Aslam et al., 2011)]. In several insect species, the activity of the ILP/IR signaling system was found to be directly modulated in relation to the nutritional and feeding state, (e.g., changes in activity or in peptide/protein/transcript levels for ILP or some other ISP components: Masumura et al., 2000; Ikeya et al., 2002; Colombani et al., 2003; Puig and Tjian, 2006; Arsic and Guerin, 2008). Other studies report that some insect ILPs are only released to signal nutrient availability under specific conditions, or following specific physiological or developmental behavioral events (e.g., only during molting or metamorphosis, although the feeding stage has already been finished) or for inducing growth under starvation conditions (Slaidina et al., 2009; Liu et al., 2010; Aslam et al., 2011).

In general, insect ILPs mainly originate from the central nervous system, but several ILPs (also) seem to be produced and released by the fat body, as well as by some other tissues (Mtioui et al., 1993; Iwami et al., 1996; Ikeya et al., 2002; Suenobu et al., 2004; Badisco et al., 2008; Okamoto et al., 2009; Nilsen et al., 2011; Okamoto et al., 2011). The function of ILPs in insect physiology has been extensively studied in the fruit fly *D. melanogaster*, which possesses (at least) eight ILPs (*Drosophila* ILPs: dILPs), some originating from the brain and other parts of the central nervous system, others from the midgut, fat body or imaginal discs (Colombani et al., 2012; Garelli et al., 2012; Nassel, 2012). It is worth noting that the central nervous system exerts the general control of the insects' body physiology, while the fat body acts as the main metabolic center (Figure 2). The fruit fly brain was found to release ILPs into the hemolymph in relation to nutritional state, although incubation of brains in the presence of nutrients did not result in ILP-release (Geminard et al., 2009). Instead, it was shown that, upon nutrient availability, the fat body releases a humoral factor that induces ILP-release from the brain cells. Since fat body-specific silencing of the Slimfast amino acid transporter or the TOR signaling resulted in decreased ILP release from the brain, as well as global growth defects and reduced PI3K signaling in peripheral tissues, it has been suggested that this humoral factor is released by the fat body in response to the presence of amino acids (Colombani et al., 2003; Geminard et al., 2009). A recent study shows that the fruit fly fat body indeed produces a factor (the cytokine-like factor Unpaired 2) that is released in response to nutrient availability. Interestingly, although Unpaired 2 has so far not been demonstrated in the hemolymph circulation *in vivo*, fat body-derived Unpaired 2 was suggested to induce ILP secretion from the fruit fly brain by acting as a humoral factor. However, the fat body Unpaired 2 expression did not seem to be dependent on nutrient-derived proteins, but rather on dietary fats and sugar. These findings suggest that different ILP release-stimulating factors might be released from the fat body in response to the presence of different nutrients (Rajan and Perrimon, 2012) and that the fat body plays an important role in sensing and signaling the “systemic” nutritional state (Figure 2).

Previous reports could already link the insect ILPs with the presence of carbohydrates. The silkworm ILP, bombyxin, is *in vivo* released from the brain in response to glucose availability (Masumura et al., 2000). Although the fruit fly brain does not seem to readily release ILPs in the presence of glucose (Geminard et al., 2009), some electrical properties of ILP-producing cells change in the presence of glucose, further suggesting a link between ILPs and carbohydrate availability (Fridell et al., 2009; Kreneisz et al., 2010). Other studies demonstrate a link with fat availability and mobilization (Banerjee et al., 2012, 2013). Although much remains to be learned about the precise regulation of insect metabolism by the ISP, it seems that this pathway is not only linked with amino acid availability, but also with fat and sugar availability, which would correspond with its presumed role of nutrient sensor.

Most studies on insect ILPs focused on ILPs originating from the central nervous system, or used systemic experimental manipulations. Because of this, the precise role of ILPs originating from non-neuronal tissues remains underexposed. A study on the mealworm beetle, *Tenebrio molitor*, suggests that the midgut might release an ILP that influences glucose catabolism in the fat body (Mtioui et al., 1993). Several reports demonstrate that, at least in some insect species, the fat body itself can also be a source of ILPs (Badisco et al., 2008; Okamoto et al., 2009; Slaidina et al., 2009; Nilsen et al., 2011; Okamoto et al., 2011; Bai et al., 2012). The fruit fly dILP 6 is specifically released from the larval fat body to stimulate imaginal disc growth, in response to starvation or in specific periods of developmentally arrested feeding (Slaidina et al., 2009), and would affect fat body metabolism (Bai et al., 2012). ILP production directly by the fat body might thus constitute a(n) (alternative) nutrient sensing system that signals the nutrient storage information that is directly available from this tissue.

Since the ISP signals the “systemic” nutritional state of the insect, it may not be surprising that this pathway influences diverse physiological processes related to the acquisition, usage and metabolism of diet-derived nutrients. Besides affecting feeding behavior (Stafford et al., 2012; Zhao and Campos, 2012) and digestion (Gulia-Nuss et al., 2011) in some species, the ISP influences diverse anabolic processes, supporting reproduction, development and growth.

## The ISP and female reproductive physiology

### Key concepts of female reproductive physiology

#### Organs associated with reproductive physiology

Key organs in the female insect reproductive physiology are the ovaries and the fat body. Typical for insect ovaries are the two lateral oviducts that join into a common oviduct. Development of the oocytes takes place in tubular structures, termed ovarioles, which merge into the lateral oviducts. During development an oocyte moves in the anterior-posterior direction within the ovariole. Consequently, the most developed oocytes (terminal oocytes) are at the base of the ovariole, near the oviduct. The number of ovarioles is largely species-specific, but may also vary within one species (Büning, 1994). In many insect species, the terminal batch of oocytes in each ovariole develops synchronically and is released into the lateral oviduct, whereupon the next batch of oocytes can start expanding. Hence, egg production is usually a cyclic process. A crucial aspect of oocyte development is the accumulation of yolk proteins that serve as a source of nutrients for the developing embryo. Vitellogenins, the yolk protein precursors, are produced by the fat body and are released into the circulating hemolymph. The oocytes take them up by means of an endocytotic mechanism, mediated by a specific vitellogenin receptor that belongs to the class of low-density lipoprotein receptors (Raikhel and Dhadialla, 1992; Tufail and Takeda, 2009). In addition to providing yolk protein precursors, the fat body also delivers the energetic substrates and the building blocks necessary for anabolic processes that are to be executed within the ovary (Figure 2).

#### The lipophilic hormones

An indispensable role in communication between and within tissues associated with reproduction is attributed to the lipophilic hormones, ecdysteroids and juvenoids. Although well known for their critical role in larval development and metamorphosis, these hormones are synthesized again in the adult stage and contribute to the production of mature eggs. [Although less documented, they also contribute to reproductive events in males.] The term “ecdysteroids” is the covering name for a group of structurally similar, cholesterol-derived, insect hormones. Ecdysone (E) and 20-hydroxy-ecdysone (20E) are the physiologically most relevant ecdysteroids (Lafont and Kooman, 2009; Lafont et al., 2012). In the adult stage, ecdysteroids are mainly synthesized in the gonads (Brown et al., 2009), although other tissues have been suggested as additional sources of ecdysteroids in some insect species (e.g., Delbecque et al., 1990; Gillot and Ismail, 1995). Ecdysteroids have autocrine and paracrine regulatory roles in ovary and oocyte development, since they control ovary morphogenesis, differentiation of germ line stem cells and development of follicle cells (Parthasarathy et al., 2010; Gancz et al., 2011; Konig et al., 2011; Ting, 2013). In addition, conjugates are stored in the eggs as an embryonic source of ecdysteroids. An endocrine regulatory role has furthermore been observed in Diptera, where circulating ecdysteroids act upon the fat body to stimulate vitellogenin synthesis (Huybrechts and De Loof, 1977) (Figure 2). The cyclic progress of female mosquito (*Aedes aegypti*) reproduction is reflected in the appearance of the fat body, each cycle proceeding through the previtellogenic stage, the vitellogenic stage and a temporary termination of vitellogenesis that is characterized by autophagy of the fat body cells. Interestingly, ecdysteroid signaling has in this organism also been shown to activate autophagy of fat body cells (Bryant and Raikhel, 2011). In addition, although the early stages of oogenesis, as well as initiation of vitellogenesis in *B. mori* require the presence of ecdysteroids, their absence seems to be necessary for completion of vitellogenesis and choriogenesis (Dhadialla et al., 1998; Swevers and Iatrou, 2009). These two examples indicate that the outcome of ecdysteroid action in reproductive physiology may be stage-dependent and species-specific.

Juvenile hormone (JH) belongs to the class of sesquiterpenoids and is produced by the *corpora allata* (CA). Like ecdysteroids, juvenoids occur in several isoforms, JH III being the most prevalent in insects (Darrouzet et al., 1997; Goodman and Granger, 2005; Kotaki et al., 2009). In many species, JH is known to stimulate vitellogenin production by the fat body, as well as vitellogenin sequestration by the growing oocytes (Figure 2) (Davey, 1981; Hartfelder, 2000; Fei et al., 2005). The latter is probably mediated by JH in two possible ways. First, JH promotes shrinkage of the follicle cells surrounding the developing oocyte and thus allows the yolk protein precursors to reach the oocyte (Tobe and Pratt, 1975). Second, some papers report on the stimulatory effect of JH analogs on the transcript levels of the vitellogenin receptor (Chen et al., 2004; Clifton and Noriega, 2012). In fact, JH has a regulatory function in many aspects of insect biology (in addition to reproduction), such as metabolism, immunity, stress tolerance and ageing, but also behavior, diapause, migration and (caste) polyphenisms, which makes that JH is also capable of indirectly influencing insect reproduction (reviewed by Simonet et al., 2004; Flatt et al., 2005; Verma, 2007). It is thus without doubt that JH is an important player in this physiological process.

### ISP-mediated control of female reproductive physiology

#### Interactions with ecdysteroid synthesis and signaling

As mentioned above, the lipophilic insect hormones, ecdysteroids and JH, regulate important aspects of insect reproductive physiology, and in this context multiple interactions with insect ISP have been reported. In addition to a regulatory activity of the ISP on the synthesis and release of the lipophilic hormones, ecdysteroids and JH were found to target some of the physiological processes that are also influenced by the ISP and, in some cases, even influence ILP levels and/or ISP activity (Figure 2).

The relationship between insect insulin signaling and ovarian ecdysteroid synthesis has mainly been investigated in dipteran species. Overall, insulin signaling seems to have a stimulatory effect on ecdysteroid production in the ovaries of these species. Fruit flies mutant in the IR indeed displayed impaired ovarian ecdysteroid synthesis (Tu et al., 2002). However, ovarian release and hemolymph levels of ecdysteroids as well as JH biosynthesis were merely not affected in homozygous Chico (the *Drosophila* IRS homolog) mutants, although oogenesis in these flies seemed to be perturbated (Richard et al., 2005). These findings might indicate a possible rescue mechanism for the Chico mutants in ecdysteroid and JH production, but not in oogenesis (see also Interactions with Juvenile Hormone Synthesis and Signaling).

The amino acids required for yolk protein production in mosquitoes are derived from a blood meal. Hence, the reproductive cycle in female mosquitoes is induced upon a positive change in the mosquito's systemic nutritional status. It had been observed that, in response to the blood meal, ecdysteroidogenic neurohormones are released (Lea and Brown, 1990). Later on, evidence was accumulating that insulin-like peptides were among them. First, a mosquito IR (MIR) was cloned from mosquito ovary mRNA and its transcript levels appeared to vary in function of the reproductive cycle (Graf et al., 1997). Second, upon a blood meal, MIR is mainly expressed in ovarian follicle cells, which are considered to be the production sites of ecdysteroids (Helbling and Graf, 1998). Third, ecdysteroid synthesis in ovaries of unfed mosquitoes could be stimulated by means of a porcine insulin treatment (Riehle and Brown, 2002). Fourth, by using specific insulin signaling inhibitors, bovine insulin-stimulated ecdysteroidogenesis was shown to act through the IR and the PI3K/PKB pathway (Riehle and Brown, 1999). And finally, an endogenous mosquito ILP (ILP3) has indeed been demonstrated to bind the MIR and to stimulate ovarian ecdysteroidogenesis (Brown et al., 2008; Wen et al., 2010). In addition to ILPs, another mosquito ecdysteroidogenic neurohormone is released upon ingestion of a blood meal. This “ovary ecdysteroidogenic hormone” (OEH) (Brown et al., 1998) displays sequence similarity to neuroparsins (Badisco et al., 2007).

Several functional interactions between the ecdysteroid pathway and the ISP have been reported in the context of growth and molting, demonstrating the complex relationships between both pathways (e.g., Orme and Leevers, 2005; Mirth and Riddiford, 2007; Francis et al., 2010; Walsh and Smith, 2011). Some reports describe molecular signal transduction components that take part in both pathways [e.g., the small GTPase Rab4b (Hou et al., 2012)], while others demonstrate functional interactions between components of both pathways, resulting in modulated activity of one of both pathways [e.g., interaction between FOXO and an ecdysone receptor coactivator (Francis et al., 2010); regulatory activity on insulin signaling by an ecdysone-repressed microRNA (Jin et al., 2012); ecdysone inhibiting insulin signaling (Colombani et al., 2005)]. Also in the regulation of insect reproductive physiology, multiple functional links between both pathways have been demonstrated. *In vitro* experiments using the yellow fever mosquito, *A. aegypti*, showed that only the combination of insulin and 20E stimulated expression of yolk protein precursors (Roy et al., 2007). Some reports describe that ecdysteroids can induce the expression of an IGF-like peptide in the silkworm *B. mori* (Okamoto et al., 2009, 2011). Interestingly, this peptide was suggested to be a growth factor for adult development, since it stimulated development of adult-specific structures [e.g., sperm duct, ejaculatory duct and several reproductive accessory glands (Okamoto et al., 2009)]. Other papers report on the nutritional status affecting ecdysone levels in adult females of the fruit fly *D. melanogaster*, resulting in effects on oogenesis and vitellogenesis (Bownes, 1989; Terashima and Bownes, 2005; Terashima et al., 2005). Whether this nutrient dependency of ecdysone levels in adult females of this species results from regulatory activity of insulin signaling in relation to nutrient availability remains to be investigated.

#### Interactions with juvenile hormone synthesis and signaling

Organisms displaying reduced insulin signaling are generally characterized by an extended life span, a phenomenon that in insects is often associated with reduced JH levels. Increased longevity in *Drosophila* IR mutants was indeed restored to wild type longevity upon JH (analog) treatment and the CA of the young adult mutants were proven to produce little JH (Tatar et al., 2001; Tatar, 2004). It has been suggested that JH deficiency upon reduced insulin signaling is not the result of impaired development of the CA tissue, but most probably relates to the disturbed neuroendocrine activation of the CA. The *Drosophila* IR is expressed in the CA, indicating a direct action of ILPs on this tissue (Belgacem and Martin, 2006). Either the ILPs are directly delivered to the CA by projections of the *corpora cardiaca* (CC) or they are received from the circulating hemolymph. It should however not be excluded that ILPs (also) act indirectly by affecting the production or activity of allatoregulatory peptides (Tu et al., 2005). As mentioned before, neither ecdysteroid nor JH levels appeared to be affected in homozygous *Drosophila* Chico mutants (Richard et al., 2005). Similarly, when studying the time-course of JH production during the first 10 days of *Drosophila* adulthood, IR mutations appeared to be more effective than Chico mutations in reduction of JH synthesis (Tu et al., 2005). It is in this context worth noting the more recent identification of the *Drosophila* adaptor protein Lnk, which appears to act in parallel to Chico in the ISP. It has been suggested that Lnk and Chico exert independent functions as well as partially overlapping functions (Werz et al., 2009). Therefore, it is not unlikely that Lnk rescues part of the defects resulting from mutations in Chico, although a role for the extended DIR isoform should also not be excluded.

Similarly as in *Drosophila*, the defects observed upon an RNAi-mediated knock-down of the IR in mosquitoes could be rescued by a JH treatment (Sim and Denlinger, 2008). It has furthermore been suggested that JH biosynthesis is dependent upon the insect's nutritional status. Hence, JH seems to act in concert with the insulin and TOR signaling pathways in regulating nutrient allocation in relation to reproductive physiology (Schal et al., 1997; Noriega, 2004; Hernandez-Martinez et al., 2007; Nouzova et al., 2011; Clifton and Noriega, 2012; Perez-Hedo et al., 2013). Further information that links the insulin/TOR signaling pathways, JH synthesis and nutrition comes from honeybee studies. A larva fed on nutrient-rich royal jelly will in normal circumstances develop into a queen bee, a process that is characterized by elevated JH titers. However, RNAi-mediated knock-down of either the IRS or TOR impeded royal jelly-fed larvae to develop into queens and resulted in the worker phenotype. Since application of JH was able to rescue the queen bee phenotype in either knock-down condition, this study offers an extra argument for a regulatory role of the ISP and TOR signaling in JH synthesis (Mutti et al., 2011). Interestingly, it was recently demonstrated in the German cockroach that insulin-mediated control of JH synthesis probably results from its inhibition of FOXO. RNAi-mediated knock-down of FOXO resulted in increased JH and vitellogenin production, even if the females had been starved. Under conditions of nutrient shortage, FOXO is suggested to translocate to the nucleus as a result of reduced ISP and, amongst others, to inhibit JH biosynthesis (Suren-Castillo et al., 2012).

In addition to ISP-mediated control of JH synthesis, some other functional interactions between the ISP and JH pathways have been described. In the beetle *Tribolium castaneum*, there is a stimulatory effect of JH on expression of some ILP genes in the fat body and brain of adult females. In addition, RNAi-based silencing of a JH biosynthesis enzyme and the JH receptor “methoprene-tolerant” caused decreased ILP gene expression (Sheng et al., 2011). Another example of a physiological process regulated by JH and ISP, is the report of Baumann et al. (2013), who show that the JH pathway and the ISP team up to stimulate lipid accumulation during tsetse fly lactation, although the actual nature of their interaction in exerting this effect is not clear yet (Attardo et al., 2012; Baumann et al., 2013).

#### Vitellogenesis

Studies in some insect species clearly demonstrate a direct stimulatory activity of ILPs, mostly in conjunction with lipophilic hormones, on vitellogenin synthesis by the fat body (Figure 2). Bovine insulin did indeed trigger yolk protein precursor production in *in vitro* mosquito fat body cultures only when it was applied together with 20E. Moreover, yolk protein precursor synthesis was impaired by supplying the medium with inhibitors of PI3K or TOR signaling (Roy et al., 2007). Similarly, *in vivo* knock-down of the MIR delayed expression of vitellogenin genes (Gulia-Nuss et al., 2011). These findings indicate that not only ovarian ecdysteroidogenesis, but also vitellogenesis, is controlled by the PI3K/PKB pathway. That control of vitellogenesis by ILPs is complex and is manifested at multiple levels has more recently also become clear by the observation that ILPs are likely to control blood meal digestion in mosquitoes. In addition to its stimulatory effect on ovarian ecdysteroidogenesis, *A. aegypti* ILP3 also stimulates trypsin-like expression in the midgut. Insulin signaling thus appears to synchronize the ecdysteroid-mediated start and progress of vitellogenesis with the availability of amino acids that are necessary for this process (Gulia-Nuss et al., 2011). In addition, the presence of amino acids is “sensed” by the TOR signaling pathway, which in turn stimulates transcription of vitellogenin genes (Hansen et al., 2004, 2005; Roy et al., 2007; Roy and Raikhel, 2011). Insulin and TOR signaling are thus both necessary in the control of vitellogenin synthesis in mosquitoes.

Similar observations have been made in the red flour beetle, *T. castaneum*, where knock-down of ISP and TOR signaling components mimicked the situation of starvation and led to severely reduced transcription of vitellogenin genes (Parthasarathy and Palli, 2011). The same study also demonstrated that vitellogenin transcript levels could not be restored in beetles mutant in the ecdysone receptor and in one of the JH biosynthesis enzymes that were re-fed after a period of starvation. These findings indicated that correctly functioning JH, ecdysteroid and insulin/TOR signaling pathways are crucial for vitellogenin gene expression. Most interestingly, silencing of JH biosynthesis and signaling components in *T. castaneum* did not only reduce ILP gene expression, but also resulted in FOXO-mediated inhibition of vitellogenin gene expression [the vitellogenin gene contains FOXO response elements] (Sheng et al., 2011). These findings show another example of direct ISP action in control of vitellogenesis by the fat body.

The stimulatory role of ILPs in vitellogenesis has also been shown in other insect species, although it remains to be proven whether they either directly or indirectly act upon the fat body. For instance, vitellogenin transcript levels were significantly reduced upon RNAi-mediated silencing of the IRP precursor transcript from the desert locust, *Schistocerca gregaria* (Badisco et al., 2011). It is worth noting that, in contrast to most other insect species, only one IRP has been identified in locust species. Moreover, the desert locust IRP is highly expressed in both brain and fat body, and expression in the latter is elevated during vitellogenesis (Badisco et al., 2008). Therefore, IRP produced by the fat body may act as a messenger that signals information about the nutrient reserves in this tissue and as an (alternative) system to sensing the locust's nutritional status contributing to the animal's metabolic control and energy homeostasis.

#### Oogenesis

In addition to controlling ecdysteroid synthesis in the ovary, which has been proven in some insect species, the ISP may also directly regulate correct progress of oogenesis within this tissue (Figure 2). A correctly functioning ISP appears to be necessary to regulate *Drosophila* egg production in response to dietary changes. Chico mutants displayed reduced proliferation of follicle stem cells and their egg chambers did not develop into the vitellogenic stage, even in the abundant presence of nutrients (Drummond-Barbosa and Spradling, 2001). It has later been demonstrated that germ line stem cell proliferation in response to nutrition is not solely controlled by insulin-dependent mechanisms, but requires other control mechanisms too. These mechanisms each act during specific phases of the cell cycle (Hsu et al., 2008). Interestingly, germ line cell division and cyst growth in *Drosophila* appear to be directly controlled by insulin derived from the central nervous system, indicating that information about the fly's nutritional status is possibly first processed by the central nervous system (LaFever and Drummond-Barbosa, 2005). Female sterility of Chico mutations appears to be autonomous to the ovary, since oocytes do not mature beyond the last previtellogenic stage although the flies display normal JH and ecdysteroid titers. Moreover, transplantation of wild type previtellogenic ovaries in the Chico mutant females resulted in normal oogenesis, indicating that the required systemic factors are present. In contrast, transplanted mutant ovaries into wild type females still displayed disturbed oogenesis. It is possible that a disturbed ISP in the ovaries leads to failure of yolk protein uptake (Richard et al., 2005). That yolk uptake by the oocytes is indeed likely to be mediated by ILPs was demonstrated in mosquitoes. *A. aegypti* ILP3, which is known to be produced in the brain, was able to rescue yolk protein uptake in decapitated females. At the moment, it is however not clear whether this effect is direct or either results from the ecdysteroidogenic activity of ILP3 (Brown et al., 2008). Similar observations have been done in *T. castaneum*, where knock-down of the IR, Chico or TOR resulted in total female sterility (no egg production) and severely reduced egg production was observed upon knock-down of Akt, PTEN, PI3K or S6K (one of the kinases phosphorylated by TOR). The observed defects that resulted from silencing these ISP components were due to impaired maturation of the primary oocytes and arrest of oocyte growth before migration of the follicles (Parthasarathy and Palli, 2011).

Female desert locusts that had been treated with IRP dsRNA were shown to have smaller terminal oocytes than the control animals, although it remains to be investigated whether this also involves a direct IRP action upon the ovary or either results from decreased vitellogenin transcription observed in the same animals (Badisco et al., 2011). Interestingly, a CC extract derived from a related locust species (*Locusta migratoria*) was capable of inducing ecdysteroid synthesis in previtellogenic ovaries of the blowfly, *Phormia regina*. There were arguments that the ecdysteroidogenic factor in this extract was likely to be an insulin-like substance (Maniere et al., 2009). Therefore, it is possible that locust IRP has ecdysteroidogenic activity on blowfly ovaries. However, it remains to be investigated whether this also holds true in locust species.

It was recently demonstrated in *Drosophila* that mutations in the IR and Chico did not exactly mimic the condition of starvation. Upon nutrient shortage, ovarian cells undergo programmed cell death and egg chambers degenerate. However, although their egg chambers developed abnormally, no programmed cell death was observed in the ovaries of IR and Chico mutants. Furthermore, these mutant flies even appeared resistant to starvation-induced programmed cell death. The ISP thus appears to be necessary for the correct response (programmed cell death) upon starvation, but it does certainly not act alone in control of this process. Mutations in TOR and S6K, on the other hand, did mimic starvation-induced programmed cell death. Most probably, the ISP and ISP-independent TOR signaling team up to mediate a correct programmed cell death response upon starvation (Pritchett and McCall, 2012).

#### Reproductive diapause

Several insect species are capable of transforming into some sort of reproductive resting stage—the so-called “reproductive diapause”—upon unfavorable environmental conditions. Reproductive diapause is in fact a type of phenotypic plasticity and is mainly elicited by alterations in temperature, photoperiod and/or food availability. It either enables an insect to survive adverse conditions or to postpone reproductive events until the environmental conditions are optimal for survival and development of the offspring. Depending on the insect species, reproductive diapause occurs in females and/or males (Pener, 1992). Since it has become clear that a crucial role in reproductive physiology is attributed to the ISP, it is not surprising that reduced insulin signaling is one of the key mechanisms controlling diapause. The low JH titer observed in diapausing insects is considered to be the causative factor and may result from reduced insulin signaling (Tatar and Yin, 2001). Diapause-like phenotypes as well as JH deficiency were indeed displayed by fruit flies mutant in the IR or Chico (Chen et al., 1996; Bohni et al., 1999; Suren-Castillo et al., 2012). A similar phenotype resulting from either ILP1 (Sim and Denlinger, 2009) or IR knock-down (Sim and Denlinger, 2008) in the mosquito *Culex pipiens* could be rescued by administration of JH. The same study pointed to a key role for FOXO, since a knockdown of this molecule prevented the mosquitoes from entering the reproductive diapause state (Sim and Denlinger, 2008). FOXO proteins are generally associated with stress resistance, determination of life span and response to starvation. They promote conservation of energy or, when necessary, even catabolic processes. Reproductively diapausing insects are characterized by lipid accumulation in the fat body (triglycerides have a higher caloric content per unit of weight than glycogen). FOXO seems to be abundantly present in the fat body of diapausing *C. pipiens* and is considered to be critical for lipid sequestration. Interestingly, JH application suppressed FOXO in diapause-destined mosquitoes, indicating that probably both the ISP and JH are capable of suppressing FOXO. This study also indicates that either FOXO and JH interact in a complex way or that their interaction is species-specific (e.g., in the German cockroach, FOXO is suggested to be an inhibitor of JH synthesis). Shortage of JH may be a causative factor for FOXO activation in mosquitoes. In addition, low JH titers in mosquitoes are suggested to reduce the fat body response to ecdysteroids and therefore to lead to fat body hypertrophy rather than vitellogenin synthesis (Sim and Denlinger, 2013).

#### Caste differentiation and division of reproductive labor

Typical for eusocial insects, such as the honeybee (*Apis mellifera*), is the division of reproductive labor within the colony. Honeybees display a pronounced type of phenotypic plasticity, which is reflected in the occurrence of two specific phenotypes—queens and workers—each optimized for the specific tasks they exert. Under normal circumstances, the colony's queen is long-lived and is the only female that reproduces, whereas the workers have a much shorter lifespan and exert other tasks, such as nursing the developing juveniles or foraging. A female will develop into a queen only when she has been fed the nutrient-rich royal jelly during the larval stages. Queen development is characterized by elevated JH titers during the fourth and fifth instars. The ISP acts also in this specific case most probably as a sensor of nutritional status and is believed to constitute the link between nutrition and developmental fate of the honeybee.

Higher transcript levels of ILPs and IRs have been observed in queen larvae when compared to worker larvae. However, this situation changes further in the developmental progress when ILP levels appear to be higher in workers (Wheeler et al., 2006; de Azevedo and Hartfelder, 2008). Knock-down of the IRS caused development of the worker phenotype even when the larvae where fed on rich diet (Wolschin et al., 2011). A complementary study aimed at blocking the nutrient sensing system by a knock-down of IRS or TOR in royal jelly-fed larvae. Knock-down of IRS and TOR (both individually and simultaneously) clearly prevented the increase in JH titers necessary for queen development and resulted in the worker phenotype (Mutti et al., 2011). It should however be noted that the ISP might not be the only signaling pathway that is at play in caste differentiation. Royalactin, a protein present in royal jelly was demonstrated to trigger several of the queen bee-related characteristics and these effects appeared to be mediated by the epidermal growth factor receptor (EGFR) signaling (Kamakura, 2011). Interestingly, in mammals, IRS has been shown to mediate EGFR signaling too (Fujioka and Ui, 2001; Fujioka et al., 2001) and similarities or interplay between the ISP and EGFR signaling would therefore not be unlikely in honeybee caste differentiation in response to a rich diet (Mutti et al., 2011).

In addition to the conserved nutrient-sensing mechanism during larval development, other species-specific ISP-related mechanisms are probably at stake in the control of egg production in reproducing queens. Indeed, the role of the ISP in control of reproduction in the adult honeybee queen appears to strongly deviate from the conserved role in most other insects. ILP transcript levels seem to be lower in adult queens than in adult workers, which could explain their extended life span (reduced insulin signaling is generally associated with increased longevity, a phenomenon that appears to be evolutionary conserved). In contrast to many other insect species, lowered ILP transcript levels (and thus likely a reduced ISP) do not result in reproductive defects in the honeybee queen (Corona et al., 2007). In addition, some other contradictions are observed in honeybees. First, although queen bees do have a higher nutritional status throughout their lifetime than workers, their adult ILP transcript levels are lower. These findings suggest that the theory of increased insulin signaling activity upon a better nutritional status does not apply to honeybees. Second, whereas JH is well-known for its stimulatory effect on vitellogenin synthesis in several insect species, it suppressed vitellogenin transcription in honeybees. Interestingly, honeybee vitellogenin, which is abundantly present in the reproducing queen, was shown to act as an anti-oxidant. This may be an alternative explanation for the increased longevity of honeybee queens (Seehuus et al., 2006). In addition, honeybee vitellogenin also appeared to affect the levels of ILPs. These findings indicated that, at least in honeybees, vitellogenin is more than just a yolk protein precursor but is likely to function as a signaling molecule too. It was therefore suggested that the ISP, JH and vitellogenin act in a complex regulatory network that regulates honeybee longevity and reproduction (Corona et al., 2007). Studies of the IRs in two ant species suggested that active insulin signaling is required for correct progress of the reproductive physiology in these social insects (Okada et al., 2010; Lu and Pietrantonio, 2011). Therefore, the somewhat contradictory role of the ISP in honeybees is probably restricted to a minority of (social) insect species.

#### Other roles of insulin in reproduction-associated processes

The production of cuticular hydrocarbons (many of which are pheromones) in *Drosophila* also seems to depend on insulin signaling. In a process that probably (also) involves the nutrient-dependent TOR signaling, the ISP stimulates transcription of the cuticular hydrocarbon synthesis enzymes. These findings illustrate that fruit flies that have a positive nutritional status and thus also a healthy and vital appearance, do probably display a higher sexual attractiveness (Kuo et al., 2012).

Only recently, light has been shed on the role of the ISP in controlling reproduction in viviparous insects. In tsetse flies, there is only one, intra-uterinely developing, larva per reproductive cycle. The developing larva is nourished by a milk-like substance secreted from a specialized gland (the so-called “milk gland”). The milk-like substance has a high lipid content, which has been derived from the mother's lipid reserves. Interestingly, lipid allocation during lactating (larvigenesis) and non-lactating (oogenesis and embryogenesis) periods is controlled by insulin and JH signaling. More specifically, JH and the ISP suppress lipolysis during the non-lactating periods (Baumann et al., 2013).

## The ISP in interaction with other hormones/pathways

As previously mentioned, the ISP influences multiple aspects of insect physiology. This pathway exerts its biological function not by acting alone, but by interacting with other (neuro)endocrine pathways. As has become clear from the sections “Interactions with ecdysteroid / juvenile hormone synthesis and signaling,” the ISP acts in concert with the lipophilic hormones to control multiple aspects of insect reproductive physiology. In most insect species, the ISP appears to have a stimulatory effect on lipophilic hormone synthesis. However, the situation appears to be more complex, since *vice versa* ecdysteroids [e.g., *B. mori* (Okamoto et al., 2009, 2011)] and JH [e.g., *T. castaneum* (Sheng et al., 2011)] have in some species been demonstrated to affect ILP expression. In addition, it is also likely that the ISP and the lipophilic hormone signaling pathways act in parallel in control of certain processes. Hence, control of insect reproductive physiology by the ISP and the lipophilic hormone pathways appears to be complex. Moreover, a species-specific and stage-dependent outcome of insulin, ecdysteroid and JH signaling should not be excluded.

Since the insect ISP is involved in the regulation of diverse physiological processes, it may also not be surprising that this pathway displays interactions with several other neuropeptide pathways. Most functional connections between the ISP and other neuropeptidergic pathways have been demonstrated in the context of nutrient homeostasis, metabolism and feeding. Indeed, several neuropeptides modulate ILP release and/or expression in the fruit fly brain [tachykinin-related peptide (Birse et al., 2011); sulfakinin (Soderberg et al., 2012); short neuropeptide F (Lee et al., 2008, 2009); and possibly also corazonin (Kapan et al., 2012)]. In other cases, the ISP itself seems to influence the expression or activity of other neuropeptide pathways. This is the case for the signaling pathways of sulfakinin (insect homolog of the vertebrate cholecystokinin), which is expressed in many of the insulin-expressing cells of the fruit fly brain (Soderberg et al., 2012), and neuropeptide F (Wu et al., 2005; Lingo et al., 2007). Because of the functional links between metabolism and reproductive physiology, these pathway interactions might (indirectly) take part in the regulation of reproductive physiology. Some reports suggest interactions between ISP and other neurohormonal pathways that have been (more directly) associated with reproductive physiology. Neuroparsins constitute a family of arthropod-specific neurohormones (Badisco et al., 2007). Based on their sequence similarity with insulin-like growth factor binding proteins (IGFBPs), neuroparsins were suggested to be potential modulators of ILP functioning, an idea that was supported by the observation of *in vitro* binding of locust neuroparsin and IRP (Badisco et al., 2008). Since some neuroparsins display anti-gonadotropic activity in locusts (Girardie et al., 1987; Badisco et al., 2011), it is possible that these act as (negative) regulators of ILP-signaling (Badisco et al., 2011). However, as it is also the case for vertebrate IGFBPs, other modes of action may occur. It is worth noting that the mosquito neuroparsin-like factor, OEH, is a gonadotropic factor and appears to act in parallel with ILP(s) (Brown et al., 1998). Some insect species do not seem to possess neuroparsin-like molecules [e.g., several *Drosophila* species (Veenstra, 2010)], although the actual occurrence of these factors remains uncertain. In *D. melanogaster* and the moth *Spodoptera frugiperda*, yet another factor with apparent sequence homology to vertebrate IGFBP has been implicated in the regulation of insulin signaling (Sloth Andersen et al., 2000; Honegger et al., 2008). This factor, named “Imaginal morphogenesis protein-Late 2” (Imp-L2), binds endogenous ILPs and in this way, modulates insulin signaling (Alic et al., 2011; Bader et al., 2013).

In addition to the above-mentioned interactions with neuropeptide and lipophilic hormone pathways, some studies suggest that the ISP in the fruit fly brain also displays functional links with specific neurotransmitter pathways [e.g., involving serotonin (Ruaud and Thummel, 2008; Luo et al., 2012), octopamine (Crocker et al., 2010; Erion et al., 2012), and γ-aminobutyric acid (Enell et al., 2010)]. However, these interactions were not studied in relation to reproductive processes, and it is therefore not clear whether they are also of importance for the control of reproductive physiology.

## The ISP and male reproductive physiology

As discussed above, many studies have already analyzed the role of the ISP as a nutrient-dependent regulator of female insect reproductive physiology. Although less documented than its role in females, the insect ISP also coordinates several aspects of male reproductive physiology according to the insect's nutritional state. Insulin signaling was found to regulate spermatogenesis in male fruit fly testes (Ueishi et al., 2009; McLeod et al., 2010; Wang et al., 2011). Besides directly inducing maintenance and proliferation of germ line stem cells in relation to nutrient availability, the ISP also affected spermatocyte growth in testes of male *D. melanogaster* (Ueishi et al., 2009; McLeod et al., 2010; Wang et al., 2011). A study in other *Drosophila* species suggested that the ISP also influences the growth of male external genitalia (Masly et al., 2011). Studies on different species of horned beetles suggested that the ISP also mediates the growth of the horns that are used by males of these species for competing with rival males, when protecting female mating partners (Emlen et al., 2012; Lavine et al., 2013). For several species of horned beetles, an interesting male reproductive dimorphism, associated with different reproductive strategies (reproductive trade-off) has been described. Larger males develop large horns for competing with other males, while small males instead choose to additionally invest in their testes and copulatory organs, in order to increase their copulation success when performing “sneak copulations” (Emlen, 1997; Tomkins and Simmons, 2000; Simmons and Emlen, 2006). This reproductive dimorphism seems to be mediated by insulin signaling, in relation to the male's nutritional status. Depending on nutrient availability and resulting body size, insulin signaling (through FOXO) regulates the growth of horns for competition or instead of testes and copulatory organs (Snell-Rood and Moczek, 2012; Lavine et al., 2013). When comparing with the literature on female insect reproductive physiology, it seems that ISP function in relation to male reproductive physiology has received little attention so far. The diversity of physiological processes affected by ISP in female insects suggests that several target processes of the ISP in adult males may remain to be identified.

## Conclusions

The current review illustrates that insulin signaling, in response to the insect's nutritional status, contributes at different levels to the control of reproductive physiology. In addition to lipophilic hormone synthesis, vitellogenesis and oogenesis, the ISP may also regulate reproductive diapause, caste development, pheromone production and even lactation physiology in viviparous insects. Energy derived from nutrition must not only be invested in development of reproductive structures, but also needs to be incorporated in the eggs as a source of nutrition for the developing embryo. Therefore, one of the most important tasks of the ISP is the allocation of energy to specific ongoing processes related to reproductive physiology. However, the ISP does not act alone in control of the aforementioned processes. It is part of a complex interaction network that also involves the lipophilic hormones and other (neuro)peptides, and in which the different components act in parallel and/or mutually interact with each other and with the available nutrients.

So far, studies relating the insect ISP to reproductive physiology have mainly been conducted in dipteran species and to a much lesser extent in other insect species. That the findings from dipteran insect studies cannot always be extrapolated to other species is nicely illustrated by the fact that some of the established relationships involving nutrition, insulin signaling and vitellogenin synthesis seem to be inverted in honeybees. Thus, a lot remains to be learned from insects belonging to non-dipteran orders.

### Conflict of interest statement

The authors declare that the research was conducted in the absence of any commercial or financial relationships that could be construed as a potential conflict of interest.

## Abbreviations

20E: 20-hydroxyecdysone
AMPK: AMP-activated protein kinase
CA: corpora allata
CC: corpora cardiaca
dILP: Drosophila insulin-like peptide
DIR: Drosophila insulin receptor
Drk: downstream of receptor kinase
E: ecdysone
EGFR: epidermal growth factor receptor
ERK: extracellular signal-regulated kinase
FOXO: forkhead-related family of transcription factors
Grb2: growth factor receptor-bound protein 2
IGF: insulin-like growth factor
IGFBP: insulin-like growth factor-binding protein
ILP: insulin-like peptide
Imp-L2: imaginal morphogenesis protein—late 2
IR: insulin receptor
IRP: insulin-related peptide
IRS: insulin receptor substrate
ISP: insulin-signaling pathway
JH: juvenile hormone
MAPK: mitogen-activated protein kinase
MEK: mitogen-activated ERK-activating kinase
MIR: mosquito insulin receptor
OEH: ovary ecdysteroid hormone
PDK: phosphoinositide-dependent protein kinase
PI3K: phosphatidylinositol-3-kinase
PIP2: phosphatidylinositol-4,5-bisphosphate
PIP3: phosphatidylinositol-3,4,5-trisphosphate
PKB: protein kinase B
PTEN: phosphatase and tensin homologue
RTK: receptor tyrosine kinase
S6K: ribosomal protein S6 kinase
SH2: Src-homology 2
Sos: Son of Sevenless
TOR: target of rapamycin
TSC: tuberous sclerosis
